# Copenhagen prodromal study III : Long term stability of self-disorders

**DOI:** 10.1192/j.eurpsy.2025.717

**Published:** 2025-08-26

**Authors:** S. Ørsted, M. G. Henriksen, J. Nordgaard

**Affiliations:** 1 Mental Health Center Amager; 2 Psychiatry Region Zealand, University of Copenhagen, Copenhagen, Denmark

## Abstract

**Introduction:**

Detailed knowledge on prognostic predictors and illness trajectories of many mental disorders, especially schizophrenia spectrum disorders, is needed towards implementation of more personalized treatment.

A potential candidate to predict prognosis and illness trajectories is self-disorders. Self-disorders are non-psychotic symptoms that seem to be present years prior to illness onset and to persist after remission from psychotic episodes. Self-disorders describe a fundamental disturbance of consciousness and can be assessed with the *Examination of Anomalous Self-Experiences* (EASE) scale.

We know from previous studies that self-disorders are non-psychotic symptoms central to schizophrenia spectrum disorders.

**Objectives:**

The purpose of this study is to examine if self-disorders are related to clinical and functional outcome as well as illness trajectories and life courses.

We will test if high levels of self-disorders at baseline predict worse outcome in terms of psychopathology, social function, recovery, more malign illness trajectories and life courses for the individual patient.

Additionally, we will examine if self-disorders remain stable over a period of 25 years.

**Methods:**

This ongoing study is a 25-year follow-up study of a cohort of 151 first-admission patients, comprising 51 patients with schizophrenia, 50 patients with schizotypal disorder, and 50 patients with other mental disorders (including bipolar disorder, major depression, anxiety, and personality disorder).

This cohort is the oldest and largest sample in the world that has been assessed for self-disorders. By combining psychosocial and psychopathological history as well as psycho-diagnostic assessments and tests at follow-up examination, with information from the Danish registers, we will obtain as complete a picture as possible of the patients and their illness trajectories and life courses. This will not only be at the point in time of the follow-up, but throughout the last 25 years and gain further knowledge about self-disorders over time. For assessments of psychopathology we will use semi-structured interview using EASE as well using OPCRIT, PANSS, GAF-S and parts of the Bonn Scale for psycho-diagnostic. Social functioning will be measured using the Personal and Social Performance scale (PSP) and GAF-F.

**Results:**

Ongoing study, results are pending.

**Conclusions:**

Ongoing study.

**Disclosure of Interest:**

None Declared

